# Down-Regulation of CIDEA Promoted Tumor Growth and Contributed to Cisplatin Resistance by Regulating the JNK-p21/Bad Signaling Pathways in Esophageal Squamous Cell Carcinoma

**DOI:** 10.3389/fonc.2020.627845

**Published:** 2021-02-03

**Authors:** Ya-Ping Gao, Lei Li, Jie Yan, Xiao-Xia Hou, Yong-Xu Jia, Zhi-Wei Chang, Xin-Yuan Guan, Yan-Ru Qin

**Affiliations:** ^1^Department of Clinical Oncology, The First Affiliated Hospital, Zhengzhou University, Zhengzhou, China; ^2^Department of Clinical Oncology, The University of Hong Kong, Hong Kong, China; ^3^Department of Clinical Oncology, The Third Peoples Hospital of Zhengzhou, Zhengzhou, China

**Keywords:** esophageal squamous cell carcinoma, cell death inducing DFF like effector A, methylation, cisplatin, apoptosis

## Abstract

Esophageal squamous cell carcinoma (ESCC) is one of the most common malignancies with poor prognosis and lack of effective targeted therapies. In this study, we investigated the tumor suppressive role of the cell death inducing DFF like effector A (CIDEA) in ESCC. Firstly, public datasets and ESCC tissue microarray analysis showed that CIDEA was frequently down-regulated at both the mRNA and protein level. This was significantly associated with low differentiation and TNM stage in ESCC, and indicated poor prognosis for ESCC patients. Bisulfite genomic sequencing (BGS) and methylation-specific PCR (MSP) analysis revealed that the down-regulation of CIDEA was associated with hypermethylation of its promoter, which was also correlated with the poor prognosis in ESCC patients. *In vitro* and *in vivo* functional studies demonstrated that CIDEA decreased cell growth, foci formation, DNA replication, and tumorigenesis in nude mice. Further study revealed that, during starvation or cisplatin induced DNA damage, CIDEA facilitated the G1-phase arrest or caspase-dependent mitochondrial apoptosis through the JNK-p21/Bad pathway. Therefore, CIDEA is a novel tumor suppressor gene that plays an important role in the development and progression of ESCC, and may provide a potential therapeutic target for patients with ESCC.

## Introduction

Esophageal squamous cell carcinoma (ESCC) is a major subtype of esophageal cancer that ranks sixth in the causes of cancer-related death all worldwide (1). The incidence and mortality of ESCC has an extremely uneven geographical distribution, as more than half of ESCC cases occur in China, particularly in Linzhou, Henan (1, 2). Although great progress has been made in early diagnosis and treatment, the 5‐year survival rate of ESCC patients is still poor (3, 4). Above all, it is essential to investigate the molecular mechanisms involved in ESCC development and progression to facilitate novel diagnostic biomarkers and candidate treatment targets specific to ESCC.

The Cancer Genome Atlas (TCGA, https://www.cancer.gov/tcga) (5, 6), a large-scale genomic dataset, provide an opportunity to better understand the biological systems of cancer. Thus, the mRNA expression and clinical survival data of ESCC in the TCGA data portal was comprehensively analyzed. Among the top 100 genes with differential expression in ESCC, only 17 genes, including cell death inducing DFF like effector A (CIDEA), were associated with prognostic significance.

CIDEA is a member of the cell death inducing DFF like effector (CIDE) family, which was initially identified in 1998 by sequence homology to the N-terminal region of the apoptotic DNA fragmentation factors 45 (DFF45) (7). DFF is a heterodimeric protein composed of DFF45 (45kDa) and DFF40 (40kDa) subunits that plays a main role in the process of DNA fragmentation during apoptosis. The apoptotic role of CIDEA was demonstrated by a study reporting that ectopic-expression of CIDEA induced DNA fragmentation and apoptosis in multiple human cell lines (7). However, the underlying mechanism of CIDE-induced apoptosis is not conclusive. It was considered to be independent of the caspase pathway, since the apoptosis induced by CIDEA could not be blocked by a pan-caspase inhibitor (7). A later study showed that apoptosis induced by CIDEA was dependent on caspase 3 activation and the release of cytochrome c from mitochondria (8, 9). In addition, CIDEA is also reported to be involved in insulin sensitivity and lipid metabolism (10–13). Mice deficient in CIDEA exhibit a lean phenotype, increased metabolic rate and reduction of lipid droplet size in the white adipose tissue. The functions of CIDEA proteins may provide a link between energy metabolism and apoptosis. In glioblastoma, CIDEA is reportedly down-regulated and is a regulator of glioma cells, where ectopic expression of CIDEA triggered apoptosis, actin cytoskeletal disruption, and cell cycle arrest (14). After a decade of study, however, the physiological role and function of CIDE proteins have not been clearly elucidated.

In the present study, we investigated the possible role of CIDEA in the development and progression of ESCC. To determine the role of CIDEA, we evaluated the expression status of CIDEA and methylation of its promoter in primary ESCC tissues and ESCC cell lines. Functional assays with CIDEA overexpressing cell lines were performed to characterize the biological effects of CIDEA in ESCC tumorigenicity. The tumor-suppressive mechanism of CIDEA and its potential as a new prognostic biomarker and therapeutic target in ESCC were also addressed.

## Materials and Methods

### Cell Lines and Clinical Specimens

A total of seven esophageal cancer cell lines were used in this study. Six of them were acquired from the German Resource Center for Biological Material (DSMZ) (Braunschweig, Germany), including KYSE30, KYSE140, KYSE150, KYSE180, KYSE410, and KYSE510. The EC109 cell line was a kind gift from Professor Tsao (The University of Hong Kong). The HEK 293-FT cells were purchased from Thermo Fisher Scientific (Waltham, MA). All cell lines used in this study underwent short tandem repeat (STR) profiling, and tested negative for mycoplasma by PCR. Cells were cultured in DMEM medium with 10% fetal bovine serum (FBS) (Gibco BRL, NY), and incubated at 37°C in a humidified atmosphere with 5% CO_2_.

All the primary ESCC tumor and non-tumor tissues, including 78 pairs for RNA extraction and 248 pairs for a tissue microarray (TMA), were collected from Linzhou Cancer Hospital (Henan, China). The patients enrolled in this study received no treatment before surgery. This study was approved by the Institutional Ethics Review Board of the First Associated Hospital (Zhengzhou University) and written informed consent form was obtained from all patients.

### ESCC Tissue Array and Immunohistochemistry (IHC)

IHC staining was performed according to the standard streptavidin-biotin-peroxidase complex method (15). Staining intensity was scored as: negative (0), weak (1), moderate (2), and strong (3). The proportion of CIDEA-positive cells was scored as 0% (0), 1–10% (1), 10–50% (2), 50–75% (3), and ≥75% (4). The IHC score was calculated by multiplying staining intensity and the proportion of positive cells. CIDEA level data was subjected to ROC curve analysis and a cutoff value of 5 was determined for CIDEA. Down-regulation of CIDEA was defined as a score ≤5.

### Quantitative Real-Time PCR Analysis (qRT-PCR) and Reverse Transcription

#### PCR Analysis (RT-PCR)

Total RNA was extracted with TRIzol^®^ reagent (Invitrogen). Reverse transcription was performed using the PrimeScript RT reagent Kit with gDNA Eraser (TaKaRa, Japan). The relative mRNA levels were determined by qRT-PCR with SYBR Green SuperMix (Roche, Basel, Switzerland) on a Roche LightCycler480. The comparative Ct method was used to calculate the relative expression of RNAs with β-Actin as an internal control. PCR amplifications were conducted with the GoTaqGreen Master mix kit (Promega Corporation, Madison, WI, USA) on a S1000 Thermal Cycler (Bio-Rad Laboratories, USA). PCR products were examined by 1.5% agarose gel electrophoresis. The primer sequences used for PCR were: CIDEA, forward, 5’-GCCGAAGAGGTCGGGAATAG-3’, and reverse, 5’-TATCCACACGTGAACCT GCC‐3’; β-Actin, forward, 5’ CATGTACGTTGCTATCCAGGC-3’, and reverse, 5’-CTCCTTAATGTCACGCACGAT-3’.

### DNA Methylation Analysis

Genomic DNA was extracted with a genomic DNA extraction kit (Tiangen, China). Purified DNA samples underwent bisulfite treatment using the EpiTect Bisulfite Kit (Qiagen, Germany). Bisulfite sequencing PCR (BSP) and Methylation-specific PCR (MSP) were performed with specific primers targeting the sequence between −400 and −250 bp of the CIDEA promoter region, where BSP showed difference in KYSE410, KYSE30 and KYSE150. Primers for methylation detection were designed with MethPrimer. The primers used for BSP were as follows: forward, TGTTTATGA-TATGGTTTTGAGAGTAG; and reverse, TATATAAATTTTAAACCCAAACCAC. The methylation-specific primers used were as follows: m1: AGCGGGTAGGAAG-TTTAGGC, m2: ATTTTAAACCCAAACCACGAAT. The unmethylation-specific primers used were as follows u1: TAGTGGGTAGGAAGTTTAGGTGT, u2: TAAAT-TTTAAACCCAAACCACAAAT.

### Establishment of CIDEA Overexpressing ESCC Cell Lines

The control plasmids and pEZ-Lv105-CIDEA were purchased from GeneCopoeia (Guangzhou, China). Their functions were confirmed by sequencing. Lentivirus-containing CIDEA was packaged in 293FT cells and stably transfected into the KYSE30 and KYSE150 cell lines with the ViraPowerTM Lentiviral Packaging Mix (Invitrogen, Carlsbad, CA), following the manufacturer’s instructions.

### Cell Proliferation Assay and Foci Formation Assay

Cell proliferation assay was performed with a CCK-8 assay kit (Dojindo, Japan), according to the manufacturer’s instructions. Briefly, cells (1 × 10^3^ cells/well) were seeded into 96-well plates and the cell growth rate was monitored for 7 consecutive days. For foci formation assay, cells (1 × 10^3^ cells/well) were seeded into six-well plates and cultured for 2 weeks. Then, cells were fixed with 75% ethanol and stained with 1% crystal violet. Finally, colonies containing with more than 50 cells were counted. Three independent assays were carried out.

### Western Blotting Analysis and Antibodies

Western blotting analysis was performed following the standard protocols (BioRad). Total proteins from tissues and cells were extracted with RIPA lysis buffer (Cell Signaling Technology) supplemented with protease inhibitor and phosphatase inhibitor. Antibodies used in this study were CIDEA (NBP1-76950, Novus), GAPDH (AM1020B, Abgent), p21 (#2947, Cell Signaling Technology), CylinD1 (#2926, Cell Signaling Technology), CDK4 (#12790, Cell Signaling Technology), p-JNK (Thr183/Tyr185) (#4668, Cell Signaling Technology), Bad (#9239, Cell Signaling Technology), Caspase9 (#9508, Cell Signaling Technology), and PARP (#9542, Cell Signaling Technology).

### EdU (5-Ethynyl-2′-deoxyuridine) Incorporation Assay

EdU incorporation assay was performed with the Cell-Light EdU Apollo567 In Vitro Kit (Ribobio, China) according the manufacturer’s instructions. Imaging was performed on an Olympus FV-1000 confocal microscope. Red nuclei EdU cells were examined by randomly counting 10 fields in the middle of the microscope slide and were expressed as a percentage of the total population. Three independent assays were carried out.

#### *In Vivo* Xenograft Assay

The control and CIDEA over-expressing cells of KYSE150 cells (5 × 10^6^) were subcutaneously injected into the left and right dorsal flanks of 4-week-old female BALB/C nude mice, which were purchased from the Guangdong Animal Center (Guangzhou, China). Tumor volumes were measured every 4 days using calipers and calculated as volume (mm^3^) = L (length)×W (width)^2^×0.5. One month later, the tumors were removed, weighed, and fixed in the formaldehyde solution for hematoxylin-eosin (HE) staining and IHC study. Animal experiments were conducted in accordance with the guidelines of the Institutional Animal Care and Use Committee.

### Cell Cycle and Cell Apoptosis Analysis

The cell cycle and cell apoptosis analyses were performed with the Cell Cycle Assay KIT (Wanleibio, China) and Annexin V FITC Apoptosis Detection Kit (Dojindo, Japan), respectively, following the manufacturer’s instructions. Cells were analyzed by a flow cytometry (CytoFlex, Beckman Coulter). Apoptosis was measured as the proportion of cells with Annexin V+/PI− and Annexin V+/PI+ fluorescence. Three independent assays were carried out.

### Mitochondrial Membrane Potential Assay

Mitochondrial membrane potential (MMP) was assessed with the fluorescent probe JC‐1 (Biyuntian, China) according to the manufacturer’s instructions. The fluorescence was analyzed by a flow cytometry (CytoFlex, Beckman-Coulter). Green (∼525 nm) fluorescence represents JC-1 monomers and red (∼590 nm) fluorescence represented JC-1 aggregates. The change of red to green fluorescence signaled a decrease in MMP.

### Statistical Analysis

SPSS software (version 23.0, Chicago, IL) and GraphPad Prism software (version 7.0, La Jolla, CA) were used for statistical analyses. A paired Student’s *t* test was performed to analyze the difference in mRNA expression between ESCC and normal tissues. Kaplan-Meier plots and log-rank tests were used for overall survival (OS) analysis and disease-free survival (DFS) analysis. Pearson’s chi-square test was used to analyze the correlation between CIDEA expression and clinicopathological parameters in ESCC. Univariate and multivariate Cox proportional hazards regression models were performed to evaluate independent prognostic factors of ESCC. Unpaired *t* test was performed to compare the significant differences between the two groups in foci formation, EdU incorporation and cell apoptosis. Data are shown as mean ± SD. *P <*0.05 was considered statistically significant.

## Results

### Down-Regulation of CIDEA was Associated With a Poor Outcome in ESCC Patients

**Figure 1:**
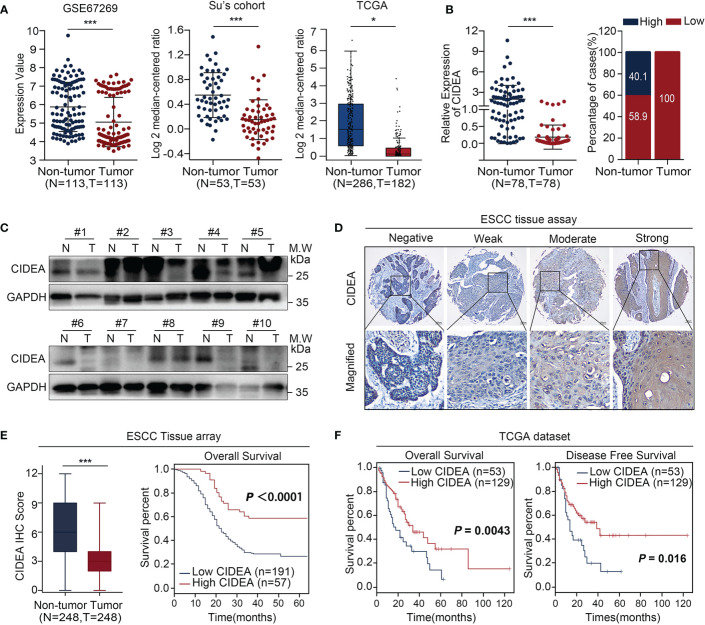
CIDEA expression was downregulated in ESCC, and low expression correlated with poor survival. **(A)** CIDEA expression was compared between ESCC tumor and normal esophageal samples derived from the GEO, Oncomine and TCGA databases. **(B)** The mRNA level of CIDEA was detected by qRT-PCR in 78 pairs of ESCC tissues and adjacent non-tumor tissues with β-actin as the internal reference control. CIDEA expression fractions in 78 ESCC tissues and adjacent non-tumor tissues. **(C)** The protein levels of CIDEA in 10 pairs of ESCC tumors (T) and corresponding normal esophageal (N) tissues were analyzed by Western blotting with GAPDH as the loading control. **(D)** Representative pictures of IHC staining of CIDEA in an ESCC tissue microarray (n = 248). Scale bars, 100 μm. **(E)** CIDEA staining scores in ESCC tumors and the corresponding non-tumor tissues (n = 248). **(E, F)** Kaplan-Meier analysis indicated that the down-regulation of CIDEA was negatively correlated with overall survival (OS) time and disease free survival (DFS) time in ESCC tissue microarray (n = 248) and TCGA dataset (n = 182). Data are presented as the mean ± SD. *P < 0.05; **P < 0.01; ***P < 0.001.

To explore the potential role of CIDEA in ESCC, the alteration of CIDEA was screened in multiple ESCC cohorts including GSE67269 (16) in GEO, Su’s (17) cohort in Oncomine, and TCGA (**Figure 1A**). Next, qRT-PCR was performed in a set of 78 pairs of ESCC and non-tumor tissues to confirm the public biostatistics. The down-regulation of CIDEA was detected in all the 78 ESCC tissues, but only observed in 46/78 (58.9%) of non-tumor tissues (**Figure 1B**). Consistently, the mRNA expression of CIDEA was significantly down-regulated in ESCC tissues.

The protein expression of CIDEA was determined by Western blotting analysis in 10 pairs of ESCC and non-tumor tissues **(****Figure 1C****)** and *via* IHC in a tissue microarray (TMA) containing 248 pairs of ESCC and non-tumor tissues **(****Figure 1D****)**. Results showed that the protein level of CIDEA was down-regulated in ESCC. In addition, Kaplan-Meier analysis based on the TMA showed that ESCC patients with low CIDEA levels had significantly shorter overall survival (OS) (*P* < 0.0001) than patients with high CIDEA levels (**Figure 1E**). Kaplan-Meier survival analysis based on the TCGA database also demonstrated the shorter OS time (*P* = 0.0043) **(****Figure 1F****)** and disease-free survival (DFS) time (*P* = 0.016) **(****Figure 1F****)** in ESCC patients with low CIDEA expression.

**Figure 2:**
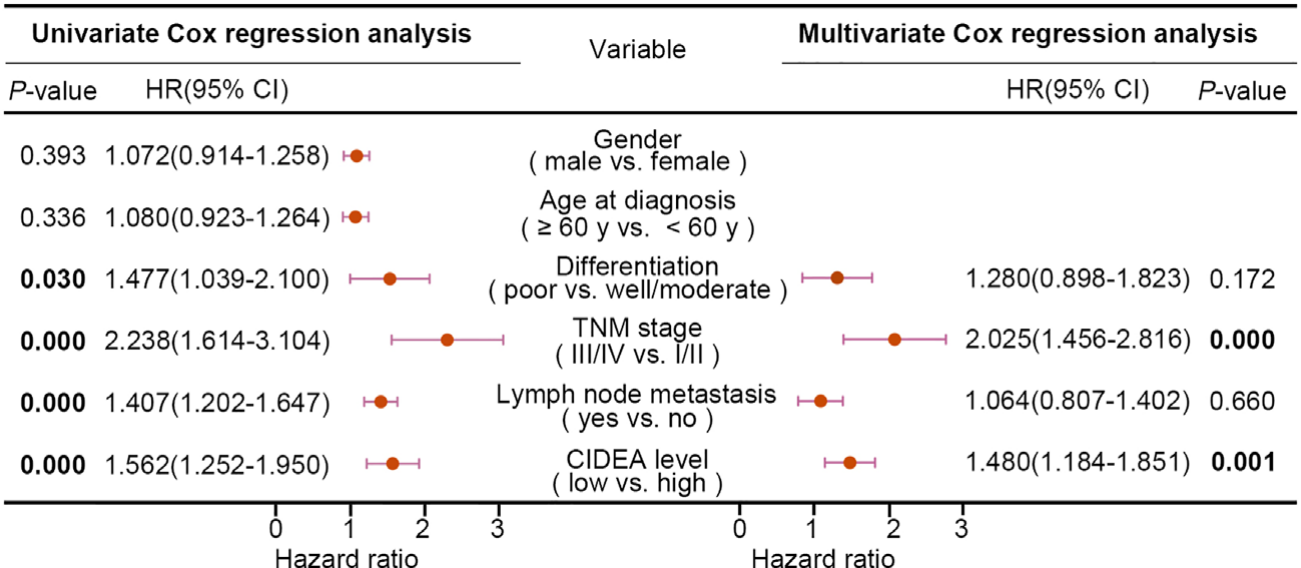
Univariate and multivariate Cox regression analyses of various factors associated with overall survival in patients with ESCC. HR, hazards ratio; CI, confidence interval. Statistical significance (*P* < 0.05) is shown in bold.

Furthermore, the association of CIDEA down regulation with clinicopathological characteristics was analyzed and summarized. CIDEA down regulation was significantly correlated with poor tumor differentiation, advanced clinical staging, and lymph node metastasis **(****Table 1****)**. Cox regression analysis using age, sex, differentiation, lymph node metastasis, and TNM stage as covariates further demonstrated that CIDEA level is an independent risk factor for OS (HR = 1.480, 95% CI = 1.184–1.851, **Figure 2**). Taken together, the results indicated that the down-regulation of CIDEA expression had a positive effect on the progression of ESCC.

**Table 1:** Associations between CIDEA levels in tumor tissues and clinicopathological characteristics in ESCC patients.

Variable	Total	expression	*P*-value
		low group, N (%)	
**Age at diagnosis**			0.99
≤60	139	107(76.98)	
>60	109	84(77.06)	
**Gender**			0.37
Male	139	110 (79.14)	
Female	109	81 (74.31)	
**Location**			
Upper	53	39 (73.85)	0.268
Middle	169	129 (76.33)	
Lower	26	123(89.46)	
**Differentiation**			
Well/moderate	180	132(73.33)	**0.025**
Poor	68	59(86.76)	
**Lymph node metastasis**			**0.0035**
N0	154	107 (69.48)	
N1	94	84 (89.36)	
**TNM stage**			**0.0028**
Early(I/II)	179	129(72.07)	
Advanced(III/IV)	69	62(89.86)	

Statistical significance (P < 0.05) is shown in bold.

### Down-Regulation of CIDEA was Associated With Aberrant Promoter Methylation

**Figure 3:**
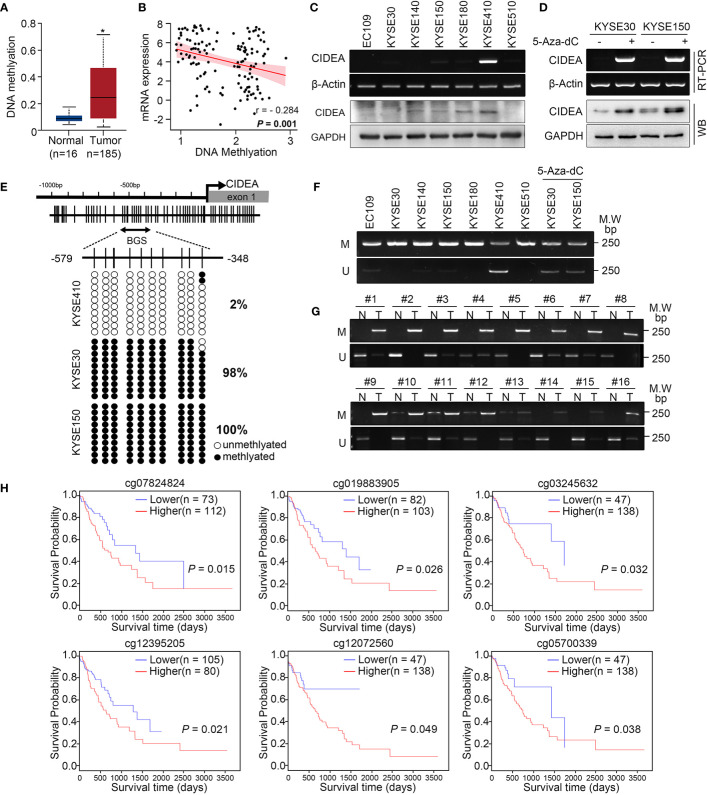
The CIDEA promoter was hyper-methylated and correlated with decreased expression of CIDEA. **(A)** The methylation level of the CIDEA promoter in TCGA ESCC samples. **(B)** Pearson’s correlation between DNA methylation and mRNA levels of CIDEA in TCGA ESCC samples. **(C)** The mRNA and protein level of CIDEA in seven ESCC cell lines were detected by RT-PCR and Western blotting with GAPDH or β-actin as loading controls. **(D)** RT-PCR and Western blotting analysis showing restored expression of CIDEA in both KYSE30 and KYSE510 cells treated with 5-AZA-DC (10 μM) for 72 h. +, 5-AZA-DC treated; -, 5-AZA-DC untreated. **(E)** The methylation status of individual CpG sites in the CIDEA promoter (between –579 and –348 bp from the transcription start site) were detected by bisulfite sequencing (BGS) in three ESCC cell lines (KYSE410, KYSE30, and KYSE150). Each row represents an individual cloned allele. The black circles show methylated CpG sites and the white circles show unmethylated CpG sites. **(F, G)** Representative methylation-specific PCR (MSP) analysis of CIDEA in ESCC cell lines and ESCC tissue samples. M, methylated allele; U, unmethylated allele. N, nontumor tissue; T, tumor tissue. **(H)** Kaplan-Meier plot showing the impact of CIDEA methylation sites of CIDEA on OS in ESCC patient as analyzed by the MethSurv webtool. Data are presented as the mean ± SD. *P < 0.05; **P < 0.01; ***P < 0.001.

To explore whether down-regulation of CIDEA is associated with aberrant DNA methylation in ESCC, the data from TCGA was analyzed. Results showed that the promoter methylation of CIDEA in ESCC (with a median beta-value of 0.257) was much higher than in normal esophageal tissues (with a median beta-value of 0.091) **(****Figure 3A****)**. The beta-value was used as a measure of methylation level, which ranges from 0 (no methylation) to 1 (complete methylation). In addition, there was a significantly negative correlation between the DNA methylation and mRNA expression of CIDEA (Pearson correlation, r =−0.284; *P* = 0.001) **(****Figure 3B****)**.

RT-PCR and Western blotting analysis indicated that silenced or down-regulated mRNA and protein expression level of CIDEA was observed in six of seven ESCC cell lines, except for KYSE410 cells **(****Figure 3C****)**. To confirm whether the methylation of CIDEA is related to its down-regulation, KYSE30 and KYSE150 cells with CIDEA low-expression were treated with 5-Aza-dC, which is a potent inhibitor of DNA methyltransferase. As shown in **Figure 3D**, after 5-Aza-dC treatment, the cellular expression of CIDEA was restored at both the mRNA and protein levels.

A potential CpG island within the upstream region of CIDEA was predicted by the public online tool MethPrimer (http://www.urogene.org/methprimer), which implied an epigenetic mechanism in the regulation of CIDEA expression. The methylation level of 10 CpG sites within the promoter region (−579 to −348) of CIDEA was analyzed in KYSE410, KYSE30, and KYSE150 cells *via* sodium bisulfite sequencing (BGS) and methylation-specific PCR (MSP). BGS analysis showed that KYSE410 cells with up-regulated CIDEA had much lower methylation levels within the region compared with KYSE30 and KYSE150 cells, in which CIDEA is down-regulated **(****Figure 3E****)**. Next, MSP was performed to validate the methylation levels within the region in the seven ESCC cell lines and a cohort of 50 pairs of ESCC and normal tissues. Compared with untreated cells, methylation was significantly reduced in KYSE30 and KYSE150 cells treated with 5-Aza-dC (**Figure 3F**). Moreover, the results showed a higher level of methylation in tumor tissues than nontumor tissues **(****Figure 3G****)**.

In addition, the relationship between DNA methylation of CIDEA and the prognostic value of each CpG site in ESCC was identified *via* MethSurv (https://biit.cs.ut.ee/methsurv/) (18). Results indicated that the methylation of cg12642717 in CIDEA was associated with the highest HR. Overall, six hyper-methylated CpG sites in CIDEA were significantly associated with poor survival, including cg07824824, cg19883905, cg03245632, cg12395205, cg12072560, and cg0570033 **(****Figure 3H****)**. Collectively, these results suggest that the down-regulation of CIDEA was closely associated with promoter hypermethylation, and the methylation of CIDEA is associated with ESCC patient survival.

### CIDEA Suppressed ESCC Tumor Growth

**Figure 4:**
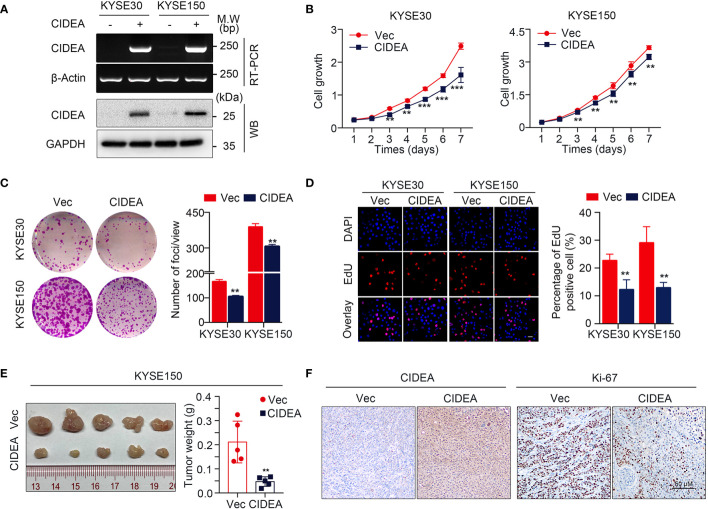
Ectopic expression of CIDEA suppressed ESCC tumor growth. **(A)** CIDEA mRNA and protein levels were detected by RT-PCR and Western blotting in CIDEA-transfected KYSE30 and KYSE150 cells. GAPDH or β-actin was used as the loading control. **(B)** Cell growth rate in CIDEA-transfected and empty vector-transfected KYSE30 and KYSE150 cells was measured by CCK8 proliferation assay for 7 consecutive days. **(C)** Representative images of decreased foci formation in monolayer culture induced by CIDEA overexpression in KYSE30 or KYSE150 cells. **(D)** Representative images (left) and quantitative statistics (right) of EdU incorporation assay showed the decreased replication of DNA in cells induced by CIDEA overexpression. Red, duplicated cells; blue, cell nucleus. Scale bars, 50 μm. **(E)** Images of xenograft tumors derived from KYSE150-transfected cells their vectors in nude mice. Tumor weights were compared between CIDEA over-expressing and control cells. **(F)** Representative IHC images of CIDEA and Ki-67 expression in xenograft tumors derived from CIDEA over-expressing and control cells. Scale bar = 50 μm. Statistical data are represented as mean ± SD. *P < 0.05; **P < 0.01; ***P < 0.001.

To explore the biological role of CIDEA in ESCC, CIDEA was over-expressed in the two ESCC cell lines KYSE30 and KYSE150 with relatively low expression of CIDEA. Ectopic expression of CIDEA was determined by RT-PCR and Western blotting **(****Figure 4A****)**. First, *in vitro* functional assays were used to measure the tumorigenicity of CIDEA. The cell growth assay and colony formation assay showed that the cell growth in the CIDEA-overexpressing KYSE30 and KYSE150 cells was significantly lower than in the control cells **(****Figures 4B, C****)**. In addition, the EdU incorporation assay also verified that the proliferative potential decreased in CIDEA-overexpressing cells **(****Figure 4D****)**. Further, the subcutaneous tumor xenograft assay in immunodeficient nude mice showed that the size and weight of xenograft tumors developed from CIDEA-overexpressing cells was significantly lower than in the controls **(****Figure 4E****)**. Furthermore, IHC staining showed that the expression of CIDEA in xenograft tumors induced by CIDEA overexpressing cells was higher, while the expression of the proliferation marker Ki-67 was lower compared to the controls **(****Figure 4F****)**.

### Ectopic Expression of CIDEA Promoted G1-Phase Arrest During Serum Starvation

**Figure 5:**
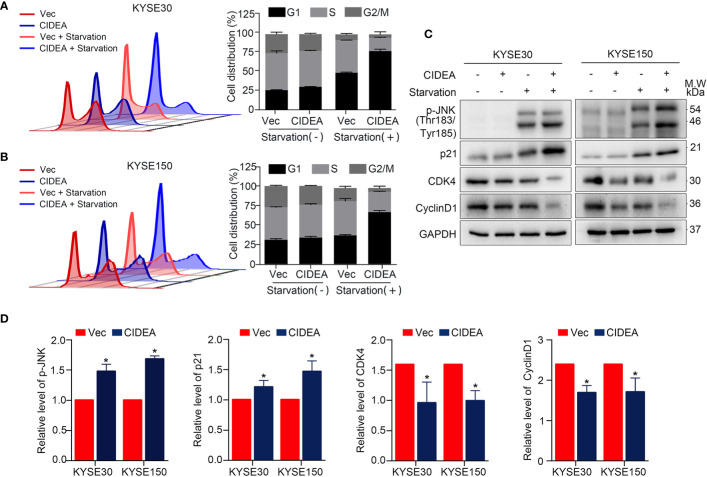
CIDEA blocked G1/S transition in ESCC cells. **(A, B)** Effect of CIDEA overexpression on the cell cycle progression of KYSE30 and KYSE150 cells. **(C)** Expression levels of cell cycle associated molecules were determined by Western blotting in KYSE30 and KYSE150 cells. GAPDH was used as a loading control. **(D)** Quantification of p-JNK, p21, CDK4, and CyclinD1 in KYSE30 and KYSE150 cells treated with serum starvation.

To explore the mechanism of CIDEA inhibition of tumor cell growth, flow cytometry was used to examine cell cycle distribution. There was no significant difference in cell distribution between CIDEA overexpressing cells and control cells under normal conditions. However, after exposure to serum-free medium for 48 h, the population of cells in the G1 phase increased in CIDEA overexpressing KYSE30 and KYSE150 compared with the control cells **(****Figures 5A, B****)**. To further characterize the mechanism by which CIDEA induced G1/S cell cycle arrest, the expression of G1/S checkpoint-related cell cycle regulators was determined by Western blotting. Under the normal conditions and serum starvation, the cellular level of p21 increased, whereas the expression of CDK4 and cyclinD1 were down-regulated in CIDEA overexpressing cells compared with control cells. In addition, during serum starvation, the level of phosphorylated JNK was greatly increased in CIDEA overexpressing cells compared with control cells **(****Figures 5C, D****)**. Collectively, these data demonstrate that CIDEA inhibits cell growth by promoting cell G1-phase arrest.

### Ectopic Expression of CIDEA Promoted Cisplatin-Induced Apoptosis Through the Caspase-Dependent Mitochondrial Pathway

**Figure 6:**
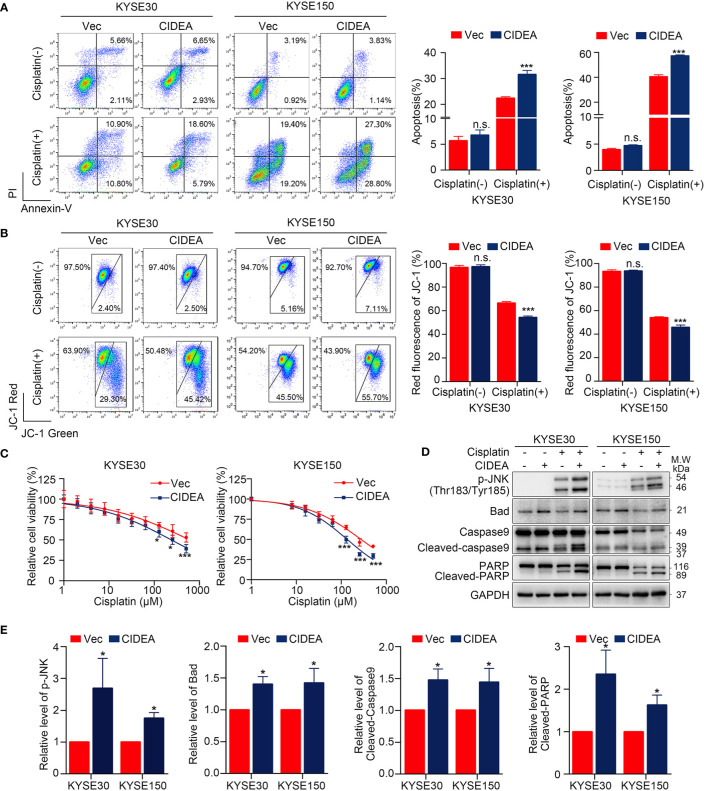
Overexpression of CIDEA promoted apoptosis in ESCC cells. **(A)** Cisplatin treated cells were stained for Annexin V/PI and analyzed FACS. The percentage of Annexin V+ cells is shown. **(B)** Mitochondrial potential was measured by flow cytometry using the JC-1 molecular probe in ESCC cells with or without cisplatin treatment. Enhanced green fluorescence ratio of cells indicated a decrease in mitochondrial potential. **(C)** Cell viability of CIDEA overexpressing cells and control cells treated with different concentrations of cisplatin. **(D)** Caspase-9, PARP, and p-JNK were detected in CIDEA overexpressing cells and control cells with or without cisplatin treatment. **(E)** Quantification of p-JNK, Bad, cleaved-Caspase9, and cleaved-PARP in KYSE30 and KYSE150 cells treated with cisplatin. Statistical data are represented as mean ± SD. *P < 0.05; **P < 0.01; ***P < 0.001; n.s., *P* > 0.05.

**Figure 7:**
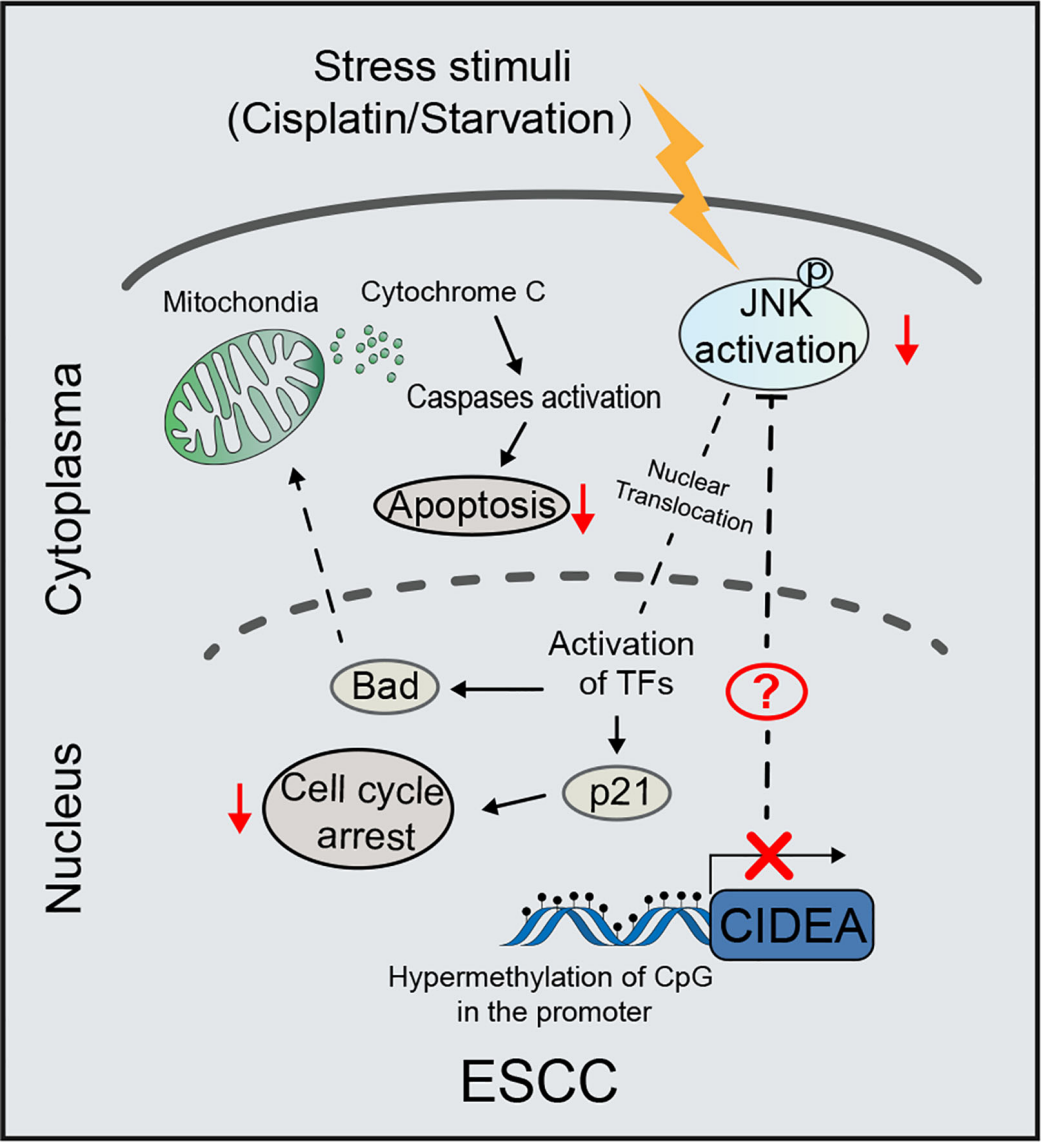
Schematic diagram illustrating the proposed tumor-suppressive mechanism of CIDEA under stress stimuli. In ESCC, the CIDEA is down-regulated due to promoter hypermethylation. When cells are stimulated with cisplatin or starvation, the activation of JNK is inhibited in ESCC with low CIDEA expression. As a result, the cisplatin-induced apoptosis and starvation-induced cell cycle arrest are suppressed. Therefore, we reason that CIDEA promotes the chemosensitivity of esophageal cancer cells to cisplatin and inhibits cell growth *via* the regulation of p-JNK.

The potential role of CIDEA in apoptosis was explored in CIDEA overexpressing and control cells with or without cisplatin treatment, as cisplatin is a drug that is widely applied in the treatment of ESCC. First, flow cytometric analysis for Annexin V/PI staining was performed. Before cisplatin treatment, the apoptosis rate was found slightly increased in CIDEA overexpressing cells compared to controls. When cells were treated with cisplatin, the apoptosis rate significantly increased in CIDEA overexpressing cells compared with control cells (**Figure 6A**). To evaluate whether the mitochondrial pathway contributed to the cisplatin-induced apoptosis in CIDEA overexpressing cells, changes in MMP were determined *via* JC-1 probe and flow cytometry. A significant decrease in MMP levels and a considerably higher ratio of JC‐1 monomers occurred in CIDEA overexpressed cells treated with cisplatin, as shown in **Figure 6B**. Next, cells were subjected to different concentrations of cisplatin for 24 h and the cell viability was determined by CCK-8 assay. As shown in **Figure 6C**, CIDEA reduced the viability of cisplatin treated cells. Finally, the expression levels of apoptosis-associated proteins were examined by Western blotting. Compared with control cells, activated caspase-9 and PARP cleavage were significantly elevated in CIDEA overexpressing cells after cisplatin treatment. Same as the cellular stress caused by starvation, during cisplatin caused DNA damage, the activation of JNK was improved in CIDEA overexpressing cells **(****Figures 6D, E****)**. Together, our results demonstrate that CIDEA, in addition to its function in inhibiting tumor cell growth, can also promote the sensitivity to cisplatin (**Figure 7**), which is an important tumor treatment modality.

## Discussion

As one of the most common types of cancer, ESCC is difficult to treat due to its high rate of malignancy, high mortality and relapse rate, and poor therapeutic response. Although great progress has been made in the early diagnosis and treatment of ESCC, the clinical outlook is still not optimistic, and the 5-year survival rate is less than 30% (19). Thus, a better understanding of esophageal cancer at both the genome and proteome level is of great significance. This would enable clinicians to accurately understand the heterogeneity of patients with esophageal cancer and conduct personalized treatment, not only avoiding unnecessary waste but improving the patient prognosis.

Although CIDEA has been identified and characterized in humans for several decades, but there remains a lack of research focused on the function and clinical significance of CIDEA in ESCC. Here, we explored the anti-oncogenic effect and the potential mechanism of CIDEA in ESCC development and progression. Upon the analysis of multiple public databases and our local cohorts, we found that CIDEA is down-regulated in ESCC both at the mRNA and protein levels. Aside from ESCC, public database results also showed that CIDEA was down-regulated in other malignant tumor types such as bladder urothelial carcinoma (BLCA), breast invasive carcinoma (BRCA), glioblastoma multiforme (GBM), head and neck squamous cell carcinoma (HNSC). Additionally, we also found that the down-regulation of CIDEA in ESCC was positively associated with tumor differentiation, TNM stage, lymph node metastasis. Furthermore, survival analysis suggested that CIDEA down-regulation indicated poor prognosis in patients with ESCC, indicating that CIDEA has the potential to serve as a novel prognostic biomarker for ESCC patients.

In cancer, aberrant methylation of CpG islands in DNA promoter regions serves as an important mechanism for the inactivation of tumor suppressor genes (20, 21). An analysis of the 1.5 kb human CIDEA promoter region showed that CpG methylation plays a key role in establishing and maintaining tissue- and cell- specific transcription of the CIDEA gene by regulating Sp1/Sp3 binding (22). This study demonstrated that the promoter hypermethylation is a possible mechanism for inactivation of CIDEA. In addition, hypermethylation of CIDEA was correlated with the poor outcome of ESCC patient. Thus, the identification of changes in CIDEA methylation during the ESCC tumorigenesis is of tremendous importance. This could contribute to early detection and new drug development for ESCC patients (23).

The tumor-suppressive function of CIDEA was demonstrated by both *in vitro* and *in vivo* assays. We showed that ectopic expression of CIDEA effectively suppressed cell proliferation, foci formation, DNA replication, and tumorigenesis in immunodeficient nude mice. Mechanism investigation found that the inhibitory effects of CIDEA on cell proliferation might be due to hindering the transition from G1-phase to S-phase. we found that, during serum starvation, the level of CKD4 and Cyclin D1 significantly decreased and p21 was up-regulated in CIDEA overexpressing cells, which facilitated the G1-phase arrest. Consistent with the previous studies that the JNK pathway can be activated by various stress stimuli like environmental stresses, DNA damage, and inflammatory cytokines (24–26), the level of p-JNK (Thr183/Tyr185) was markedly elevated in CIDEA overexpressing cells during serum starvation. Besides, the relationship between CIDEA and starvation-related signaling pathways required further experiments to clarify. In summary, we showed that during serum starvation, CIDEA facilitated the phosphorylation and activation of JNK, which in turn led to the activation of nuclear transcription factors. Thus, the transcript level of p21 gene was elevated and cells were arrested in the G1-phase.

Due to the low screening rate and the absence of obvious symptoms in the early stage of ESCC, 80–90% of ESCC cases are diagnosed as advanced ESCC, resulting in a missed opportunity for radical surgery. Platinum-based chemoradiotherapy is the standard treatment for patients with advanced and postoperative recurrent esophageal cancer. However, the chemoresistance is a key factor in the low effectiveness of treatment. Considering the pro-apoptotic role of CIDEA, it is essential to explore whether CIDEA can predict the therapeutic effect of cisplatin in ESCC patient. Our study showed that, the cisplatin-induced apoptosis, which is mainly regulated by the mitochondrial apoptosis pathway, was promoted by CIDEA. This was shown as detectable activation of caspase-9 and an obvious decrease in MMP. In response to cisplatin induced DNA damage, the activation of JNK facilitated by CIDEA promoted the transcription of pro-apoptotic protein (25, 27), including Bad. In addition, the genetic dependency of CIDEA was analyzed in the Cancer Dependency Map (DepMap) (http://depmap.org/portal) (28), which indicated a moderate dependency of CIDEA for esophageal cancer cell survival. Therefore, there should be some other factors to facilitate the tumor suppressor function of CIDEA, which required further experiments to elucidate. Collectively, we provide evidences of an important role for CIDEA in elevating sensitivity of ESCC cells to cisplatin-induced apoptosis, which suggests a potential role for CIDEA in predicting cisplatin response.

In summary, we report that CIDEA was frequently downregulated in ESCC and functions as a tumor-suppressor by regulating ESCC proliferation and apoptosis through the JNK-p21/Bad pathway. Importantly, a better understanding of the role of CIDEA may provide a novel biomarker for predicting the therapeutic effect of cisplatin and survival for patients with ESCC.

## Data Availability Statement

The original contributions presented in the study are included in the article/supplementary materials. Further inquiries can be directed to the corresponding author.

## Ethics Statement

The studies involving human participants were reviewed and approved by Institutional Ethics Review Board of the First Associated Hospital (Zhengzhou University). The patients/participants provided their written informed consent to participate in this study. The animal study was reviewed and approved by Institutional Animal Care and Use Committee, Sun Yat-sen University Cancer Center.

## Author Contributions

Y-PG, LL: acquisition, analysis, and interpretation of data. Y-PG: drafting of the manuscript. JY, X-XH, Y-XJ, X-YG, and Z-WC: technical and material support; Y-RQ: study design and supervision. All authors contributed to the article and approved the submitted version.

## Funding

This work was supported by the National Natural Science Foundation of China (81772554, 8187111604, 82072604 and 82072738), the Basic and Applied Basic Research Foundation of Guangdong Province (2019A1515110660).

## Conflict of Interest

The authors declare that the research was conducted in the absence of any commercial or financial relationships that could be construed as a potential conflict of interest.
